# Long-Lasting Muscle Thinning Induced by Infrared Irradiation Specialized With Wavelengths and Contact Cooling: A Preliminary Report

**Published:** 2010-05-28

**Authors:** Yohei Tanaka, Kiyoshi Matsuo, Shunsuke Yuzuriha

**Affiliations:** Department of Plastic and Reconstructive Surgery, Shinshu University School of Medicine, Matsumoto, Nagano 390-8621, Japan

## Abstract

**Objective:** Infrared (IR) irradiation specialized with wavelengths and contact cooling increases the amount of water in the dermis to protect the subcutaneous tissues against IR damage; thus, it is applied to smooth forehead wrinkles. However, this treatment consistently induces brow ptosis. Therefore, we investigated whether IR irradiation induces muscle thinning. **Methods:** Rat central back tissues were irradiated with the specialized IR device. Histological evaluation was performed on sagittal slices that included skin, panniculus carnosus, and deep muscles. **Results:** Significant reductions in panniculus carnosus thickness were observed between controls and irradiated tissues at postirradiation day 30 (P30), P60, P90, and P180; however, no reduction was observed in nonirradiated controls from days 0 to 180. No significant changes were observed in the trunk muscle over time. From day 0, dermal thickness was significantly reduced at P90 and P180; however, no difference was observed between P180 and nonirradiated controls at day 180. DNA degradation consistent with apoptosis was detected in the panniculus carnosus at P7 and P30. **Conclusions:** We found that IR irradiation induced long-lasting superficial muscle thinning, probably by a kind of apoptosis. The panniculus carnosus is equivalent to the superficial facial muscles of humans; thus, the changes observed here reflected those in the frontalis muscle that resulted in brow ptosis. The IR device used in this study simulated solar IR radiation that reaches the skin. Therefore, exposure to solar IR radiation may cause thinning of the superficial facial muscles. This should be prevented with sunscreen that protects against IR radiation.

We previously reported that infrared (IR) irradiation specialized with wavelengths and contact cooling penetrated the skin and smoothed wrinkles.^1^,^2^ Infrared irradiation caused an increase in the amount of water retained in the dermis by inducing the expression of collagen, elastin, and water-binding proteins, and this protected the subcutaneous tissues against IR damage. We have also reported that patients with aponeurotic blepharoptosis showed an involuntary increase in reflexive contraction of the frontalis muscles that produced forehead wrinkles.^3^ After IR irradiation was applied to smooth the forehead wrinkles, we consistently observed weakened contraction of the frontalis muscles, resulting in brow ptosis (Fig 1).

On the basis of these experiences, we hypothesized that IR irradiation specialized with wavelengths and contact cooling may induce muscle thinning. To test this hypothesis, we used this specialized IR device to irradiate the central back tissues of rats.

## MATERIALS AND METHODS

### Infrared device

**Figure 2:**
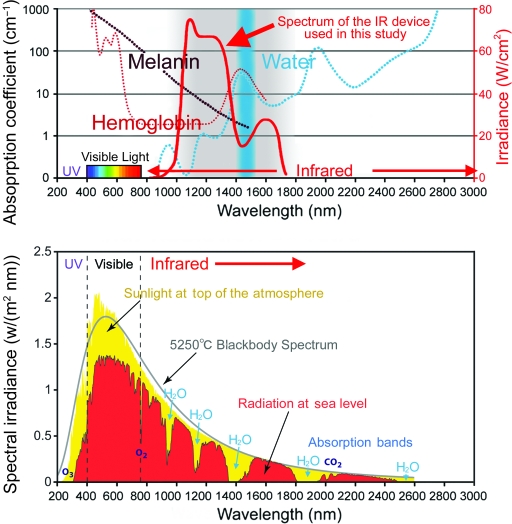
Medical and solar infrared (IR) radiation. (*Above*) Medical IR irradiation. This graph shows the absorption coefficients of melanin, hemoglobin, and water. The IR device used in this study emitted a spectrum of IR from 1100 to 1800 nm (bold red) with a filter that blocked wavelengths between 1400 and 1500 nm (blue belt), which are strongly absorbed by water and hemoglobin. (*Below*) Solar IR radiation. This graph shows the radiation spectrum for the direct light both at the top of the earth's atmosphere (yellow) and at sea level (red). The sun produces light with a distribution similar to that expected from a 5250°C blackbody (gray), which is approximately the sun's surface temperature. As light passes through the atmosphere, some is absorbed by gases with specific absorption bands (blue). These curves are based on the American Society for Testing and Materials Terrestrial Reference Spectra, which are standards adopted by the photovoltaic industry to ensure consistent test conditions and are similar to the light expected in North America. Regions for ultraviolet, visible, and IR light are indicated.

Infrared irradiation was obtained from a broadband IR source (Titan; Cutera, Brisbane, Calif). The device emitted IR spectrum in a range from 1100 to 1800 nm, with filtering to remove wavelengths between 1400 and 1500 nm (Fig 2, *above*). This delivered IR irradiation without the wavelengths that are strongly absorbed by water and hemoglobin and allowed safe delivery of IR energy deep into the tissue. The system delivered energy with a fluence range from 5/to 56 J/cm^2^, using continuous-energy single-radiation pulses of 4 to 10 seconds. The sapphire contact-cooling tip was set to a fixed temperature of 20°C to provide contact cooling. Pre-, parallel, and postcooling of the superficial layers accomplished through a temperature-controlled sapphire window further prevented excessive superficial heating.

### Animals

Male Wistar rats (*Rattus norvegicus albinus*) were housed in a temperature-controlled environment under a 12-hour light-dark cycle with free access to water and standard rat chow. Treatments were performed under anaesthesia. Samples were taken from the median region of the back, including skin, subcutaneous tissue, muscle (panniculus carnosus and trunk muscle), and spinous process. The study was approved by our institutional ethics committee for animal experiments.

### Infrared irradiation

Thirty-two male Wistar rats were either irradiated (*n* = 24) or received no treatment (controls; *n* = 8). The backs of irradiated rats were subjected to 3 rounds of irradiation doses at 40 J/cm^2^ on days 0, 7, and 14. A round of irradiation consisted of 2 passes of IR irradiation.

### Histological investigation

More than 200 specimens were obtained from 32 rats for histological examination. Samples were taken from 24 rats immediately and at 7, 30, 60, 90, and 180 days after the final dose of radiation (postirradiation day 0 [P0], P7, P30, P60, P90, and P180, respectively). Control samples were taken before and 180 days after irradiation (controls at day 0 and controls at day 180). Specimens were fixed in 20% neutral buffered formalin and processed for paraffin embedding. They were then serially sectioned in the sagittal plane (thickness = 3–4 µm). Specimens were evaluated by hematoxylin and eosin staining, Azan-Mallory staining, and the transferase-mediated dUTP nick-end labeling (TUNEL) technique. The thickness of the panniculus carnosus and dermis was evaluated from digital photographs. Images were scanned and quantified in 5 representative fields per section and then averaged to obtain a final score. The sections were photographed under a BIOREVO BZ-9000 microscope (Keyence, Osaka, Japan). The digital photographs were processed with Adobe Photoshop (Adobe, San Jose, Calif).

### Statistical analyses

The difference among the groups at each time point was examined for statistical significance by the Mann-Whitney *U* test. *P* < .05 was taken to indicate statistical significance. Data are presented as means ± standard deviation.

## RESULTS

**Figure 3:**
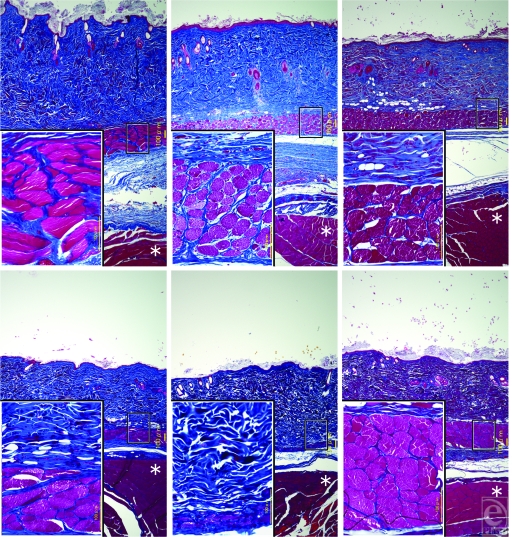
Histological changes after infrared (IR) irradiation evaluated by Azan-Mallory staining. (*Above*, *left*) Controls at day 0. (*Above*, *center*) Seven days after final-dose irradiation (P7). (*Above*, *right*) P30. (*Below*, *left*) P90. (*Below*, *center*) P180. (*Below*, *right*) Controls at day 180. A representative part of the panniculus carnosus enclosed in the smaller box of the same size is enlarged into the larger box of the same size. The asterisk (*) indicates the trunk muscle. Scale bars = 100 µm.

**Figure 4:**
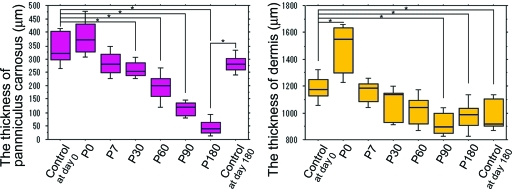
Mean changes in the thickness of the panniculus carnosus (*left*) and the dermis (*right*). Data represents the means ± SD. Control specimens: *n* = 8; irradiated specimens: *n* = 24, for each time point after infrared irradiation (P7–P180). Significant differences are indicated (*: *P* < .05).

The thickness of the panniculus carnosus decreased steadily over a 6-month period (Figs 3 and 4, *left*). Significant decreases in thickness were observed at P30, P60, P90, or P180 compared with controls (*P* < .05). No changes were observed between controls at day 0 and controls at day 180 (*P* = .1745). No significant changes were observed in the trunk muscle over time (Fig 3).

The thickness of the dermis decreased moderately over a 6-month period (Figs 3 and 4, *right*). Significant decreases in thickness were observed at P90 and P180 and in controls at day 180 compared with controls at day 0 (*P* < .05). In contrast, no changes were observed between P180 and controls at day 180 (*P* = .8345).

The thickness of the panniculus carnosus and the dermis increased temporarily at P0 because of mild swelling after IR irradiation (Fig 4).

**Figure 5:**
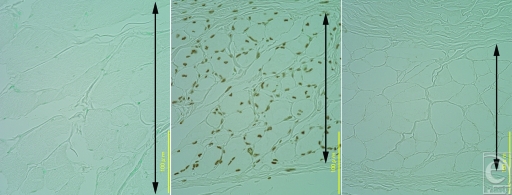
Changes after infrared irradiation were evaluated with the TUNEL technique. (*Left*) Controls at day 0. (*Center*) Seven days after irradiation (P7), the TUNEL signal was positive in the panniculus carnosus. (*Right*) Sixty days after irradiation (P60), the TUNEL signal was negative. The arrow indicates the panniculus carnosus.

The TUNEL evaluation was positive in the panniculus carnosus densely at P7 (Fig 5, *center*) and sparsely at P30 but negative at P60 (Fig 5, *right*), P90, and P180 as well as in controls at day 0 (Fig 5, *left*) and controls at day 180. The TUNEL evaluation was negative in the trunk muscle over time.

## DISCUSSION

The results suggested that IR irradiation specialized with wavelengths and contact cooling could induce long-lasting superficial muscle thinning, probably by a kind of apoptosis. A significant decrease in the thickness of panniculus carnosus was not observed between controls at day 0 and controls at day 180 but was observed between controls at day 180 and P180 (Fig 4, *left*); thus, the thinning appeared to be induced by IR irradiation and not by intrinsic aging. In contrast, a significant difference in the thickness of the dermis was not observed between P180 and controls at day 180 (Fig 5, *right*); thus, the thinning of the dermis appeared to be induced by intrinsic aging. On the other hand, no significant changes were induced in the trunk muscle; thus, the IR irradiation used in this study appeared not to penetrate to the depth of the trunk muscle.

Although the thinning of the panniculus carnosus lasted for 180 days (Fig 4, *left*), we could not confirm more significant histological changes with a longer follow-up time because of the death of rats. We previously reported that in the rat dermis, the proliferation stage lasted for about 1 month and the maturation stage began at day 45.^1^ In human tissue, the proliferation stage appeared to last for about 1 to 3 months and the maturation stage began at around 2 to 3 months.^2^ Thus, the human healing period is about 2 to 3 times longer than that in rats.^2^ In this study, we histologically examined biopsy specimens taken up to P180. Six months appeared to be a sufficiently long time, because it extrapolated to roughly 1 to 1.5 years in human tissue.^2^

At P7 and P30, the TUNEL evaluation was positive in the panniculus carnosus (Fig 5, *center*). Evidence of apoptosis has previously been based on the identification of membrane blebbing, cell shrinkage, and nuclear changes. In contrast, TUNEL identifies DNA fragmentation and can be positive for either apoptosis or necrosis.^4^ In this study, we did not detect evidence of necrosis, including inflammation and hyperplasia of fibroblasts and/or lymphocytes, or necrotic tissue were not apparently detected in P7 and P30 tissues (Fig. 3, *above*, *center* and *above*, *right*); thus, the positive TUNEL evaluation suggested that IR irradiation induced a kind of apoptosis and not necrosis.

Infrared devices without a water filter or contact cooling have been used in previous studies to evaluate photobiological effects on the human body. However, with these treatments, so much energy is absorbed in the superficial layers of skin that only limited IR energy can be delivered to deeper tissues. Many studies have elucidated the influence of superficial tissues. For example, wavelength selection directly influences target selection and penetration depth (Fig 2, *above*). Wavelengths below 1100 nm are absorbed preferentially by melanin in the superficial layers of the skin. Wavelengths between 1400 and 1500 nm and those above 1850 nm are absorbed heavily by water in the superficial layers of the skin, which results in heating and can lead to painful sensations and burns.^5^ An IR device with a water filter can penetrate the superficial layers of the skin. This facilitates investigation of the influence of IR on deeper tissues. Infrared radiation from the sun is selectively filtered by atmospheric water^6^,^7^ (Fig 2, *below*); thus, most IR radiation that reaches the earth's surface readily penetrates the superficial layers of the skin.^6^ Therefore, an IR device with a water filter reflects the natural situation and it allows evaluation of solar IR radiation that reaches the skin. In this study, we used an IR device that emitted a spectrum of IR irradiation from 1100 to 1800 nm with a filter that excluded wavelengths between 1400 and 1500 nm, which are strongly absorbed by water and hemoglobin (Fig 2, *above*). Filtering out the wavelengths below 1100 nm, around 1450 nm, and above 1850 nm enabled the delivery of IR irradiation to deeper tissues^8^ and also simulated solar IR radiation that reaches the skin on the earth's surface (Fig 2). However, in reality, both solar IR radiation with an atmospheric water filter and the IR device with a water filter used in this study increase the skin surface temperature and induce perspiration and blood vessel dilation that causes the absorption of IR radiation by water and hemoglobin. To counter this effect, in this study, we used contact cooling to reduce the skin surface temperature and thereby reduce perspiration and blood vessel dilation. These specific wavelengths and the cooling system enabled IR irradiation to penetrate the skin surface without pain or epidermal burns.^8^,^9^ This resulted in dermal thickening for wrinkle treatment but, unfortunately, also caused unexpected muscle thinning.

The panniculus carnosus is a striated muscle situated deep into the panniculus adiposus within the superficial fascia that surrounds the entire trunk of animals.^10^ This muscle causes skin-twitching movements^11^ and is an integral part of the blood supply to the skin.^12^ The panniculus carnosus is rich in hemoglobin and myoglobin, which readily absorb IR radiation; thus, this muscle will be particularly damaged by IR irradiation compared with the dermis. The superficial muscles in humans include the frontalis, orbicularis oculi, and platysma muscles in the head and neck region. These are continuous with the superficial muscular aponeurotic system and are equivalent to the discrete panniculus carnosus muscle in animals. On the other hand, the superficial muscular aponeurotic system sends several thin muscle extensions to the dermis.^13^ IR irradiation might cause long-lasting thinning of the superficial facial muscles and the muscle extensions to the dermis and thus lead to facial skin ptosis. Additional factors that are thought to contribute brow ptosis include the gradual loss of forehead skin elasticity and a reduction in the tone of the frontalis muscles.^14^,^15^ Infrared irradiation for smoothing forehead wrinkles also caused brow ptosis. Similarly, spending a lot of time in the sun might cause IR exposure that results in facial skin ptosis because of long-lasting thinning of the superficial facial muscles.

Sunlight that reaches the human skin contains solar energy composed of 6.8% ultraviolet (UV) light, 38.9% visible light, and 54.3% IR radiation (Fig 2, *below*).^16^ The total incident solar energy at sea level in North America is 0.0747 W/cm^2^.^7^ One standard forehead IR irradiation consisted of about 100 shots at 40 J/cm^2^, equivalent to the energy of approximately 4.37 hours of sunbathing in North America (Fig 2, *below*). Therefore, long-term continual exposure to incident solar IR radiation may affect the superficial muscles and the dermis. Both UV and visible light radiation are attenuated by melanin^6^; solar IR radiation with an atmospheric water filter is attenuated by thick water-containing dermis. Thus, skin with sparse melanin and a thin dermis might allow IR radiation to penetrate deeper into human tissue than skin with dense melanin and a thick dermis. Although UV blockers are often used to prevent skin damage from UV exposure, sunscreens that protect from IR radiation should also be used to prevent damage to deeper tissues.

It should be noted that this is a preliminary study based on experiments in limited number of animals and over a limited period of time. Further studies are required to test various skin types, other species, including humans, longer posttreatment periods, and other light wavelengths.

## CONCLUSIONS

Infrared irradiation specialized with wavelengths and contact cooling induces long-lasting superficial muscle thinning, probably by a kind of apoptosis. Solar IR radiation may also cause muscle thinning in sun-exposed areas of the body; therefore, exposed skin should be protected with sunscreens that block IR radiation to prevent facial skin ptosis. In contrast, IR irradiation has the potential to selectively thin and weaken dystonic or hypertrophic muscles of the superficial facial muscles.

## Acknowledgments

We thank Mr Robert Rohde (GlobalWarmingArt.com) for precise data and the file for Figure 2, *below*; Mr Ikuo Matsuyama for histological staining; and the members of Cutera, Inc, for technical information related to the IR device and helpful comments.

## Figures and Tables

**Figure 1:**
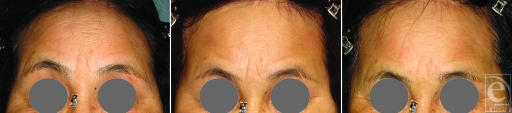
Photographs of a representative patient with forehead wrinkles treated with infrared (IR) irradiation. (*Left*) Before irradiation, a 72-year-old Japanese woman exhibited forehead wrinkles because of an involuntary reflexive contraction of the frontalis muscles that elevated the brows on primary gaze. Forty shots of irradiation were applied at 32 J/cm^2^ without topical anesthesia. (*Center*) Seven days after IR irradiation, forehead wrinkles improved, probably due to edematous changes. (*Right*) Six months after irradiation, although the forehead wrinkles were smoothed, the brows sagged, probably due to frontalis muscle thinning.
